# Population-specificity of human DNA methylation

**DOI:** 10.1186/gb-2012-13-2-r8

**Published:** 2012-02-09

**Authors:** Hunter B Fraser, Lucia L Lam, Sarah M Neumann, Michael S Kobor

**Affiliations:** 1 Department of Biology, Stanford University, Stanford, CA 94305, USA; 2 Department of Medical Genetics, University of British Columbia, Vancouver, British Columbia, V6T 1Z3, Canada; 3 Centre for Molecular Medicine and Therapeutics, Child and Family Research Institute, Vancouver, British Columbia V5Z 4H4, Canada

## Abstract

**Background:**

Ethnic differences in human DNA methylation have been shown for a number of CpG sites, but the genome-wide patterns and extent of these differences are largely unknown. In addition, whether the genetic control of polymorphic DNA methylation is population-specific has not been investigated.

**Results:**

Here we measure DNA methylation near the transcription start sites of over 14, 000 genes in 180 cell lines derived from one African and one European population. We find population-specific patterns of DNA methylation at over a third of all genes. Furthermore, although the methylation at over a thousand CpG sites is heritable, these heritabilities also differ between populations, suggesting extensive divergence in the genetic control of DNA methylation. In support of this, genetic mapping of DNA methylation reveals that most of the population specificity can be explained by divergence in allele frequencies between populations, and that there is little overlap in genetic associations between populations. These population-specific genetic associations are supported by the patterns of DNA methylation in several hundred brain samples, suggesting that they hold *in vivo *and across tissues.

**Conclusions:**

These results suggest that DNA methylation is highly divergent between populations, and that this divergence may be due in large part to a combination of differences in allele frequencies and complex epistasis or gene × environment interactions.

## Background

In multicellular organisms, the great diversity of cell types is maintained by mitotically heritable differences in gene expression, which are in part regulated by epigenetic mechanisms [1]. These include histone modifications, histone variants, RNA-based mechanisms, and DNA methylation [2]. The latter is perhaps the best understood component of the epigenetic machinery [3] and in somatic cells occurs almost exclusively on cytosine residues in the context of CpG dinucleotides [4]. While CpGs are underrepresented across the human genome, they are enriched at the majority of gene promoters, forming regions known as CpG islands that can regulate the expression of neighboring genes [4]. DNA methylation is not only closely linked to tissue-specific gene expression, but also to a number of intriguing biological phenomena such as X-chromosome inactivation in females, allele-specific expression of imprinted genes, aging, and cancer [5].

An emerging aspect of epigenetics is its role at the interface between the environment and the genome [6]. Although DNA methylation is a very stable epigenetic mark, numerous environmental influences have been associated with variation in DNA methylation as well as other epigenetic marks [2,6]. These include nutritional factors, exposure to environmental pollutants, and social environment. It is this plasticity that underlies much of the potential contribution of DNA methylation to multifactorial diseases and complex phenotypes [7]. However, the fundamental biology of the epigenome poses some challenges to testing this attractive concept. For example, most primary material available from human populations consists of mixtures of different cell types with distinct epigenomes, making it difficult to specifically assess the association of epigenetic changes with environmental exposure and phenotype. To address the role of epigenetics in common disease, it is important to understand the nature of epigenetic variation in the context of genetically well-characterized pure cell populations.

Recent advances in high-throughput technologies for measuring DNA methylation have allowed the patterns of methylation to be characterized throughout the human genome [8-15]. Comparing these results between twins has revealed that methylation at some CpG sites can be heritable [14,15], and combining them with genotype data has led to the discovery of hundreds of methylation-associated SNPs, or 'mSNPs', in brain tissue [11,12] as well as cell lines [13]. However, the question of whether the effects of mSNPs on DNA methylation levels and heritability differ between human populations has not been addressed. Quantifying such population specificity is important for our understanding of the genetic architecture of the epigenome, as well as its plasticity during human evolution.

## Results

To compare DNA methylation between human populations, we utilized lymphoblastoid cell lines (LCLs) from the HapMap project [16], which have been extensively genotyped and previously employed to study the population specificity of gene expression levels [17-19]. Although LCLs can acquire changes in gene expression and DNA methylation during transformation and cell culture [20,21], it has been shown that the inter-individual variation - which is what is relevant for the current work - is nearly always conserved (at least for gene expression) [21]. Our initial study set consisted of 30 family trios (mother/father/offspring) of Northern European ancestry (abbreviated CEU), and 30 trios of Yoruban (West African) ancestry (abbreviated YRI). These 180 cell lines were grown in identical conditions and their genomic DNA was subjected to quantitative bead-array-based DNA methylation analysis at 27, 578 CpG sites near the transcription start sites of 14, 495 genes (Materials and methods). Although an average of approximately two CpG sites near each transcription start site does not directly measure most of the methylation in regulatory regions, the fact that sites separated by under approximately 1 kb show highly correlated methylation [9,10] suggests that our data may actually capture the majority of methylation information near transcription start sites - similar to the effect of linkage disequilibrium (LD) between genetic variants in genome-wide association studies (though there is no guarantee that the most relevant sites will be in 'methylation LD' with the CpG sites we measure). The 1, 092 sites on the × and Y chromosomes were excluded from all analyses to eliminate gender effects, leaving 26, 486 autosomal sites in 13, 890 genes (in which no significant sex specificity was observed; Figure S1 in Additional file 1).

**Figure 1:**
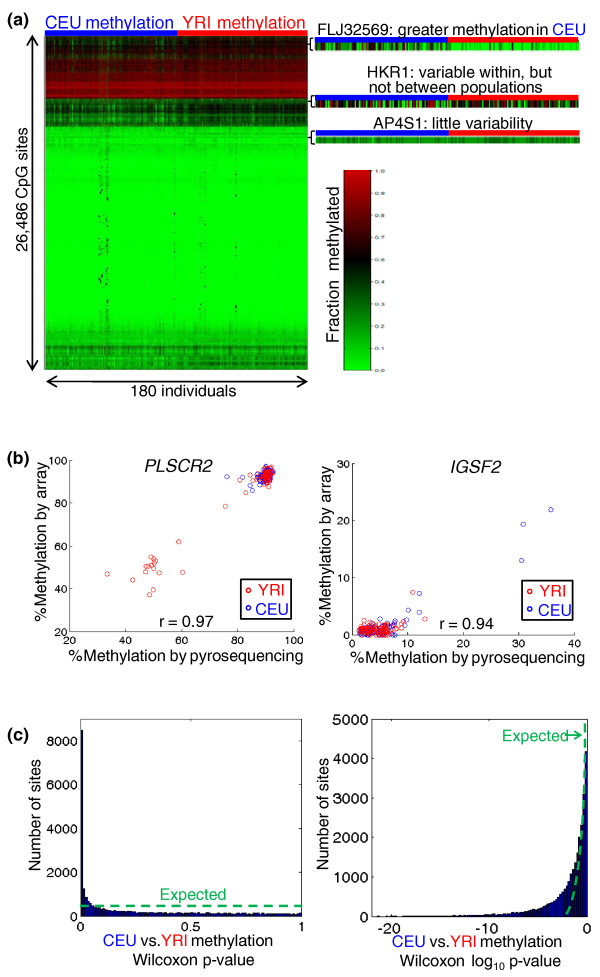
**Population-specificity of DNA methylation**. **(a) **Heatmap of the clustered methylation data set. Three representative cases are magnified: a site with a clear population difference; a site showing within- but not between-population variability; and a site with little variability within or between populations. **(b) **We performed pyrosequencing as an independent means to measure methylation of two CpG sites (*IGSF2*, chromosome 1, base 117345939; *PLSCR2*, chromosome 3, base 147696535) in our 180 samples. The agreement validates the accuracy of our microarray data. **(c) **The methylation of many sites differs between CEU and YRI. We performed the nonparametric Wilcoxon test to identify CpG sites differing in methylation between populations. The *P*-values are skewed towards small values, as shown by comparing to the expected uniform distribution on either a linear (left) or log (right) scale.

The resulting data revealed a wide range of within-population variability in the methylation of individual CpG sites (Figure 1a), consistent with previous work [11-13]. Across all sites, the average correlation of methylation profiles between individuals (mean *r*^2 ^= 0.78 for CEU, 0.86 for YRI) was far lower than that of technical replicates (*r*^2 ^> 0.99 for all six replicate pairs), indicating that most of the variability was biological, and not technical. In addition, we replicated results for two variable sites in all 180 samples by pyrosequencing bisulfite-treated DNA. This showed excellent concordance with our array-based results (*r*^2 ^= 0.88 for *IGSF2 *and 0.94 for *PLSCR2*; Figure 1b), suggesting that the array data provide accurate quantification of DNA methylation levels.

In addition to the variation within each population, we observed extensive differences in the DNA methylation patterns between populations (for example, *FLJ32569 *in Figure 1a). To quantify this population specificity, we calculated the number of CpG sites with methylation differing between populations, using the nonparametric Wilcoxon test. We found a substantial fraction differing between the populations (Figure 1c): at nominal *P *< 0.01, 8, 475 sites differed between populations (32.0% of sites; false discovery rate (FDR) = 3.1%), and 5, 654 sites remained significant at *P *< 0.001 (21.4% of sites; FDR = 0.5%; Figure S2 in Additional file 1). Thus, the methylation of approximately 30% of the CpG sites we studied - representing over a third of the genes assayed - differed between populations (this degree of population specificity is similar to that of gene expression levels in the same cell lines; Figure S3 in Additional file 1). However, these population-level differences tended to be small in magnitude, with only 1, 033 sites (3.9%) differing by an average of over 10% methylation, and 3, 695 sites (14.0%) differing by over 5%. Perhaps because of their small magnitudes, differences in DNA methylation explained very little of the variation in gene expression levels between populations that has been previously reported [17-19] (Supplemental text and Figure S4 in Additional file 1), consistent with previous findings that inter-individual variation in DNA methylation explains almost none of the variation in gene expression [12,13].

**Figure 2:**
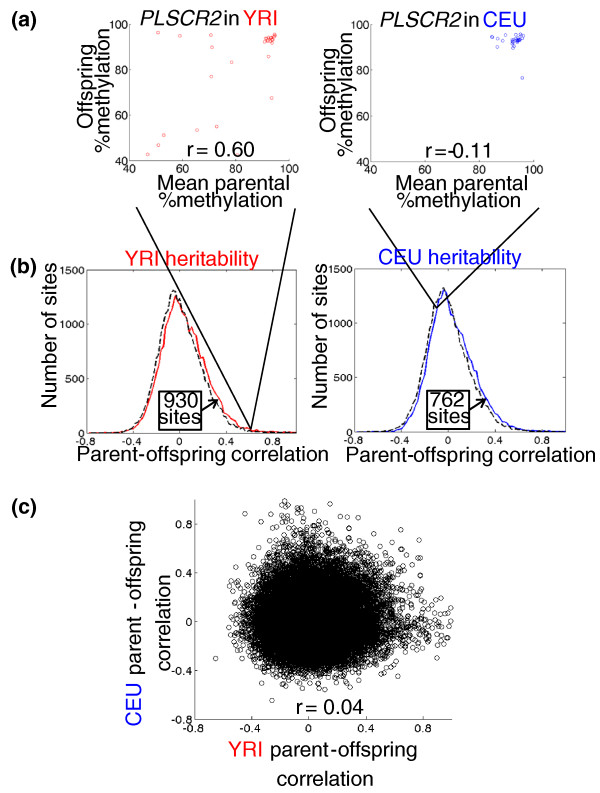
**Population specificity of DNA methylation heritability**. **(a) **An example of a CpG site (near *PLSCR2*: chromosome 3, base 147696535) whose methylation is heritable in YRI, but not CEU, as assessed by the similarity of average parental methylation to their offspring methylation (each point represents one family trio). **(b) **Histograms comparing the observed distribution of per-site heritabilities to a typical randomized distribution (numbers in the text are based on 1, 000 randomizations; Materials and methods). The greater number of sites at high heritabilities in the real data compared to random (arrows) is an estimate of the number of heritable sites we can detect in each population. **(c) **No similarity between heritabilities in each population (Pearson's *r*^2 ^= 0.002; each point is a CpG site).

These subtle but extensive epigenetic differences between populations could have genetic or environmental underpinnings - or a combination of both. To assess the role of both common and rare genetic variants in determining DNA methylation patterns, we estimated the contribution of additive genetic variation (known as narrow-sense heritability, or *h*^2^) to the methylation of each CpG site in each population by measuring the correlation in methylation levels between parents and their offspring (Figure 2a; Materials and methods). We observed heritable methylation at approximately 762 CpG sites in CEU and 930 sites in YRI (Figure 2b), suggesting that genetic control of polymorphic methylation is fairly common - though slightly less heritable than gene expression levels in the same cell lines (Figure S5 in Additional file 1). Given our limited power to detect weakly heritable DNA methylation, these numbers are likely to be substantial underestimates of the true extent of heritability.

Considering the overall genetic similarity among human populations [16,22], we expected the patterns of heritability in CEU and YRI to be similar. Surprisingly, we found almost no correlation between them (*r*^2 ^= 0.002; Figure 2c). This is similar to agreement in *h*^2 ^for gene expression levels in the same cell lines (Figure S6 in Additional file 1). We did not find any evidence for complex inheritance patterns - such as dominance, maternal-biased, or paternal-biased inheritance of DNA methylation - that could affect heritability (Supplemental text in Additional file 1).

Differences in heritability between populations could have many causes. *h*^2 ^is defined as the ratio of a trait's additive genetic variance to its total variance in a population; factors that can affect this ratio include changes in the additive genetic variance (for example, differing allele frequencies), non-additive (gene × gene, or GxG) genetic variance, environmental variance, and gene × environment (GxE) interaction variance [23]. In addition, limited statistical power could restrict the accuracy of our heritability estimates (Supplemental text and Figure S7 in Additional file 1). Although we were not able to rule out any of these potential factors, the extensive DNA sequence data available for these samples do allow us to test the contributions of two types of divergence that may contribute to the population-specific DNA methylation levels, and their heritabilities.

One type of divergence that may affect DNA methylation levels and heritabilities is a difference in the CEU/YRI allele frequencies at genetic variants that influence methylation. In particular, lower minor allele frequency at such a variant reduces the population-level genetic variation affecting a site's methylation, thus reducing *h*^2^. To test how much of our observed population specificity can be explained in this way, we first identified the 'local' SNP (within 100 kb of the CpG) most strongly associated with each CpG's methylation across all 180 samples from both populations (although genetic associations in ethnically heterogeneous cohorts such as this can reflect population stratification, it is appropriate for our current goal). We then included this single SNP genotype in a multiple regression analysis to assess whether genotype or population was a stronger predictor of methylation at each site. Among the 5, 654 CpG sites differing between populations at Wilcoxon *P *< 0.001 (discussed above), we found that 3, 131 (55.4%) were more strongly associated with a local SNP genotype than with population, implying that common (and likely *cis*-acting) genetic variants can explain over half of the population specificity we observed. This result also indicates that most of the population specificity is unlikely to be due to any type of cell line artifacts, since these would not correlate with individual SNP genotypes.

The second type of divergence we tested concerned complex GxG or GxE interactions: if a genetic variant is present in two populations, but affects DNA methylation in only one, then that variant must genetically interact with other variants and/or the environment. Such interactions can decrease heritability by increasing the population-level variance in DNA methylation (the denominator of *h*^2^) without affecting the additive genetic variance (the numerator). To perform this analysis, we needed to identify SNPs associated with the methylation of individual CpG sites separately in each population, and then compare the lists to one another.

Three previous studies of genome-wide DNA methylation have mapped SNPs whose genotype correlates with the methylation of a CpG site, termed 'mSNPs' [11-13]. Because mSNPs are highly enriched close to their target CpG sites [11-13], we performed a 'local' association analysis between methylation at each CpG site with all HapMap SNPs within 100 kb, separately for each population. These local mSNP associations can arise from either true (likely *cis*-acting) genetic associations, or genetic variants that disrupt hybridization of the bead-array probes in some individuals, leading to spurious associations (analogous to issues in eQTL mapping [24]). Using recent and essentially complete catalogs of common genetic variants in each [22], we identified all probes overlapping variants present in the 1000 Genomes samples (2, 734 probes in CEU, and 3, 923 probes in YRI; Table S1 in Additional file 1). We observed a 2.6-fold higher frequency of mSNPs for these probes compared to probes not disrupted by SNPs, implying a high rate of spurious associations (re-analysis of previously reported brain mSNPs [11,12] suggests a similarly high rate of spurious associations in those studies). Therefore, we removed these probes from our analysis (these sites did not have a higher level of heritability or population differentiation, so were not excluded from those analyses; Supplemental text and Figure S8 in Additional file 1).

**Figure 3:**
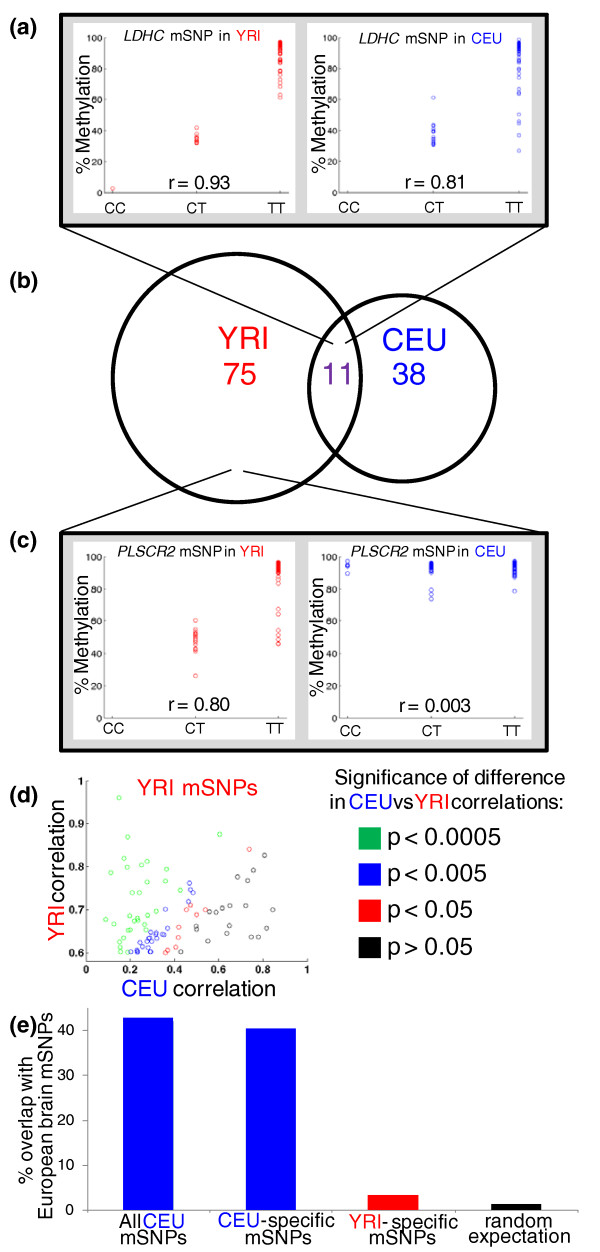
**Population specificity of mSNPs**. **(a) **An example of an mSNP (between a CpG site near *LDHC *(chromosome 11, base 18390591), and rs2643856) that is found in both YRI and CEU. In both cases the T allele is associated with higher methylation. **(b) **Venn diagram of the overlap among CpG sites associated with an mSNP in YRI and/or CEU. Five CEU sites and eight YRI sites were excluded from the overlap analysis because they overlapped a SNP in the other population. **(c) **Example of an mSNP (between a CpG site near *PLSCR2 *(chromosome 3, base 147696535) and rs12489924) that is found in YRI but not CEU. No other SNPs in CEU within 100 kb of the CpG are associated with methylation at the site (*r *< 0.25 for all), indicating that the difference is unlikely to be due to differing LD between rs12489924 and the causal variant. **(d) **Scatter plot of all 86 YRI mSNPs, showing the strongest association found for that site in each population. Points are colored according to the significance of the difference in the associations within each population; most mSNP association strengths are significantly (*P *< 0.005) different between populations. The same plot for CEU mSNPs is shown in Figure S10 in Additional file 1. **(e) **Overlap of LCL mSNPs with brain mSNPs from two studies of European populations (similar to CEU). Both all CEU mSNPs and CEU-specific mSNPs show similar overlap of 40 to 42%, which is thus a minimum estimate for the extent of mSNPs shared between LCLs and brain. However, YRI-specific mSNPs show only 3.2% overlap, not significantly different from the 1.2% expected from any random set of CpG sites.

After excluding the potentially problematic probes, we identified 49 mSNPs in CEU and 86 in YRI (genotype versus methylation level *r *> 0.6; FDR of 37% and 28%, respectively), each explaining 36 to 92% of the variance in DNA methylation at the associated site (Figure 3a). We note that these numbers are not directly comparable to previous studies [11,12] that included CpG probes that may contain SNPs, since including probes overlapping SNPs in our analysis increases the number of (apparent) mSNPs while decreasing the FDR. Restricting the CpG sites to only those with heritable methylation (*h*^2 ^> 0.2) decreased the FDR substantially (24 mSNPs at 8.6% FDR in CEU; 55 mSNPs at 4.7% FDR in YRI), providing a high-confidence list of mSNPs (Table 1; Table S2 in Additional file 1), as well as evidence supporting our heritability estimates in each population. Our high-confidence YRI mSNP list overlapped the mSNPs from a previous study of YRI LCL mSNPs [13] over 50-fold more than expected by chance (Supplemental text in Additional file 1). The vast majority of our mSNPs did not coincide with eSNPs (SNPs associated with gene expression levels; Supplemental text in Additional file 1), in agreement with previous work [13], suggesting that most do not impact gene expression levels in standard LCL culture conditions. None of these mSNPs affected methylation in known imprinted regions, and there was no enrichment for Gene Ontology categories or KEGG (Kyoto Encyclopedia of Genes and Genomes) pathways among the genes associated with either population's mSNPs.

**Table 1:** High-confidence mSNPs in CEU

Gene	Chromosome	CpG position	mSNP	Perccentage CEU variance explained	Perccentage YRI variance explained	CEU *h*^2^	YRI *h*^2^	Brain mSNP?
*TTC13*	1	229182620	rs7545429	71.3	49.0	0.41	0.64	No
*MGC3207*	19	13736014	rs371671	68.8	27.2	0.60	0.35	Yes
*PPP4R2*	3	73128376	rs9816164	66.7	43.2	0.51	0.23	Yes
*LDHC*	11	18390591	rs11601413	65.4	86.5	0.55	0.68	Yes
*RNF186*	1	20015084	rs3806308	65.1	68.3	0.41	0.50	No
*FLJ32569*	1	204085874	rs823080	58.5	**4.5**	0.28	0.05	Yes
*NDUFAF2*	5	60275337	rs162244	57.4	62.6	0.26	0.49	No
*PCGF3*	4	689950	rs2242234	57.2	**19.9**	0.47	-0.10	No
*LTA*	6	31648435	rs2516390	55.9	40.5	0.48	0.24	No
*IGSF2*	1	117345939	rs12130298	52.6	**10.0**	0.96	-0.19	No
*GSTM5*	1	110056139	rs4970776	52.4	**12.1**	0.55	0.14	Yes
*FLJ32569*	1	204085802	rs823080	50.4	**3.7**	0.49	0.08	Yes
*ASCIZ*	16	79627243	rs16954698	47.8	**9.6**	0.24	-0.12	No
*TACSTD2*	1	58815787	rs1109896	42.2	50.4	0.29	0.49	No
*HLA-C*	6	31347299	rs6457375	42.1	44.0	0.24	0.61	Yes
*HLA-DRB5*	6	32606582	rs9271586	42.0	28.2	0.32	0.42	No
*LYCAT*	2	30523367	rs829650	40.8	52.4	0.75	0.64	Yes
*PARK2*	6	163069159	rs13218900	40.4	41.6	0.21	0.03	No
*ITPR1*	3	4510075	rs304075	39.4	**7.6**	0.21	-0.07	No
*PSMD5*	9	122644335	rs12343516	39.4	35.1	0.53	0.11	Yes
*BTN3A2*	6	26472772	rs2393667	38.1	**14.9**	0.22	0.31	Yes
*RAPGEF3*	12	46439111	rs3759407	37.2	**6.8**	0.71	-0.17	No
*FAM83A*	8	124264314	rs16898095	36.3	76.5	0.27	0.71	No
*CRIP2*	14	105011436	rs4983346	36.1	**3.6**	0.46	0.04	No

The 24 mSNP-CpG site pairs where > 36% of the variance in CEU methylation is explained by the mSNP genotype, and *h*^2 ^> 0.2. When more than one SNP was tied for the strongest association (due to perfect LD), one was chosen randomly. The YRI association strength is for the top local (within 100 kb) mSNP association for the same CpG site. In bold are YRI associations that explain < 20% of the variance in YRI methylation, indicating a high-confidence set of CEU-specific associations. For brain mSNPs, the intersection of *cis*-acting mSNP lists used by the authors of each original study [11,12] was used. YRI mSNPs are listed in Table S2 in Additional file 1.

To test our mSNP mapping accuracy, we performed bisulfite Sanger sequencing at one mSNP locus (*RNF186*; Table 1) on 55 individual DNA molecules from six samples (three CEU and three YRI; Figure S9 in Additional file 1). Each individual's average methylation level at a particular CpG site (cg09195271) agreed with our array-based results (*r*^2 ^= 0.74), recapitulating the association between this site's methylation and the genotype of a nearby SNP (rs3806308): individuals with a CC genotype had the lowest average methylation (4/27 DNA molecules methylated = 14.8%), CT was intermediate (5/18 = 27.8% methylated), and TT had the highest (8/10 = 80% methylated). Interestingly, the methylation at six additional CpG sites in between rs3806308 and the target CpG did not correlate with the SNP genotype, indicating site-specific control of methylation, and not a more general regional effect.

Comparing our complete catalogs of mSNPs from each population, we found little overlap between them, or in the DNA methylation sites associated with mSNPs: only 11 CpG sites (8.9% of the mSNP-associated sites) were present in both of our medium-confidence lists (Figure 3a-c). This lack of overlap parallels the extensive population specificity of both methylation levels (Figure 1c) and their heritabilities (Figure 2c). Sites with population-specific mSNPs also tended to have population-specific heritabilities (Table 1, entries in bold; and see *PLSCR2 *in Figure 2a and 3b), suggesting that the mSNPs we detect are a major source of the heritability of their target sites' methylation.

Three factors could contribute to a lack of overlap between mSNPs from each population: low power, differing LD/allele frequencies, and true population-specific effects of genetic variation on methylation. We found that neither low power nor differing LD/allele frequencies could account for most of the population specificity we observed (Supplemental text in Additional file 1), suggesting that many mSNPs exert population-specific effects on DNA methylation. Such population specificity can only be explained by interactions between the mSNPs and other genetic variants, and/or the environment (see Discussion).

Comparing our mSNP catalogs to previously reported mSNPs from brain allows us to test the generality of the observed population specificity in an independent cohort and tissue. Among our CEU mSNPs, 42% (10/24; Figure 3e) were previously observed in both of two brain mSNP catalogs that utilized cohorts of European ancestry [11,12] (Table 1), indicating that these associations are shared across tissues. A similar fraction (4/10, 40%; Figure 3e; Table 1, entries in bold) of the subset of high-confidence mSNPs observed only in CEU (not YRI) were also seen in brain. A key prediction of our results is that mSNPs found only in YRI should not be observed in the European brain samples if they are truly population specific. In support of this, only 1/32 (3.1%; Figure 3e; Table S1 in Additional file 1) of YRI-specific mSNPs were seen in European brain (not significantly different than the 1.2% expected by chance). This lack of overlap is unlikely to be due to potential artifacts of long-term cell culture, since the CEU cell lines are decades older than the YRI, which would tend to act against the trend we observed. Therefore, we conclude that the population specificity we discovered is recapitulated *in vivo*, as well as across tissues.

## Discussion

Our results demonstrate extensive population specificity in DNA methylation profiles near transcription start sites. We observed these differences at three levels: the extent of DNA methylation, its heritability, and its association with specific genetic variants (mSNPs). We attribute most of these differences to two main factors: population-specific allele frequencies of genetic variants affecting DNA methylation, and complex GxG or GxE interactions.

Although *in vitro *artifacts are always a concern when using cell lines - and in particular LCLs, which have been shown to have some methylation differences compared to blood [20,21] - our results are unlikely to be driven by these effects, for three main reasons. First, unlike some previous studies of population-level differences in these cell lines [17,25], we processed samples in a randomized design, to eliminate the possibility of batch effects influencing our estimates of population specificity. Second, we found most of the population-specific DNA methylation to be explained by local genetic variants, ruling out any type of cell line artifact as an alternative explanation. Third, and most importantly, our population-specific mSNPs are supported by comparison to two studies of brain mSNPs in cohorts of European ancestry: 40% of our CEU-specific mSNPs overlap with both of these previous studies, whereas only 3.1% of YRI-specific mSNPs do, despite our expectation that the much older CEU LCLs would be more likely to have accumulated abnormalities in DNA methylation [20]. Together, these lines of evidence strongly suggest that our results apply *in vivo *and across tissues.

A variant that is present in two populations, but affects DNA methylation in only one, can only be explained by complex genetic interactions. These interactions could involve the environment (GxE), epistasis with other variants (GxG), or both. For example, some genetic variants have an observable effect on DNA methylation only in the presence of a sufficient quantity of methyl donors [26], which could differ between Yorubans and European-Americans as a result of diet or other factors (though methylation differences due to GxE interactions would have to be preserved during the creation and culturing of the LCLs). Even with such interactions causing differentiation between populations, genetic effects could be entirely additive within populations, consistent with our observation of heritable DNA methylation at many sites.

Divergence in the genetic underpinnings of DNA methylation (as evidenced by the population-specific mSNPs) would be expected to result in differing heritabilities and methylation levels, consistent with our results. Although we cannot provide an accurate estimate of exactly how much of the population-specific DNA methylation we observed is due to population-specific mSNPs, it is likely to be a substantial fraction once mSNPs of small effect (which could not be detected here due to our limited sample size) are accounted for.

## Conclusions

As DNA methylation is an important epigenetic modification, affecting a wide range of diseases and other phenotypes [1-7], our finding that genetic or environmental interactions likely affect most mSNPs - and thus may also explain a substantial portion of the population specificity of DNA methylation levels, and their heritabilities - underscores the complex interplay of factors that influence epigenetic modifications. Further characterization of these factors will be critical for our understanding of the epigenome.

## Materials and methods

### Genome-wide DNA methylation analysis

Genomic DNA was purchased from the Coriell Institute. DNA concentration and purity were assessed spectrophotometrically using a NanoDrop ND-1000 (Thermo Scientific, Waltham, MA, USA). After random ordering of all samples, 1 μg of genomic DNA from each sample was bisulfite-converted using the EZ-96 DNA Methylation Kit (Zymo Research, Irvine, CA, USA) as per Illumina's Infinium specific protocol. Bisulfite converted DNA was then quantified by NanoDrop and concentrated to higher than 50 ng/μl using a Speedvac.

Quantitative DNA methylation measurements of bisulfite-treated genomic DNA were performed with the Infinium HumanMethylation27 BeadChip assay (Illumina, San Diego, CA, USA), using experimental procedures recommended by the manufacturer. Briefly, 200 ng of bisulfite-converted DNA was whole-genome amplified, fragmented by an enzymatic process and hybridized to BeadChip arrays. Two oligonucleotide probes interrogated each CpG site, one probe with sequences targeting methylated DNA and the other containing sequences targeting unmethylated DNA. After extension with DNP-labeled and biotin-labeled dNTP, each array was stained with Cy5 labeled anti-DNP antibodies and Cy3 labeled streptavidin and scanned with the Illumina iScan on a two-color channel to detect Cy3 labeled probes on the green channel and Cy5 labeled probes on the red channel. Using the Illumina GenomeStudio software package, methylation levels (β values) were then calculated by dividing the methylated probe signal intensity by the sum of methylated and unmethylated probe signal intensities. β values range from 0 (completely unmethylated) to 1 (fully methylated) and provide a quantitative readout of relative DNA methylation for each CpG site within the cell population being interrogated. This method was highly reproducible, as technical replicates across different runs had *r *> 0.996. All samples passed internal controls included on the HumanMethylation27 arrays, including controls for array background, hybridization quality, target specificity and bisulfite conversion. Furthermore, all samples passed our quality control check of having fewer than 5% of sites with either detection *P*-value < 0.05 or fewer than five beads being present on the array for a particular CpG site. Cluster analysis also indicated the absence of any outlier samples. Raw data have been deposited in the Gene Expression Omnibus database under accession number [GSE27146].

Samples from both populations were run together in a randomized order to avoid confounding batch effects with population differences. In order to test for the presence of batch effects, we tested whether the DNA methylation profiles of samples run in either the same batch number (1 to 4) or well number (1 to 96) were more similar to each other than expected by chance. Neither batch number nor well number was predictive of profile similarity (comparing correlation coefficients within batches or wells to all sample correlations, Wilcoxon *P *= 0.79 and 0.64, respectively), indicating the lack of any detectable batch effects.

Several steps were applied for normalization of β values across the subjects. First, average background intensity, as measured by negative background probes present on the array, was subtracted from the raw intensities to adjust for varying background signals across different samples. This background adjustment was done separately for raw data from the green and red channels to adjust for Cy3 and Cy5 differences. All negative intensities were assigned values of zero before further normalizations were performed. To minimize batch effects across different sets of arrays, background adjusted raw data from both channels were quantile normalized separately. Applying the same formula used by GenomeStudio, average β values were then recalculated using background subtracted and quantile normalized intensities of methylated probes divided by the sum of normalized intensities from unmethylated and methylated probes.

### Pyrosequencing

DNA methylation of the promoter regions of *PLSCR2 *and *IGSF2 *containing specific CpG loci under the control of mSNPs were confirmed using bisulfite pyrosequencing. Genomic DNA (750 ng) was bisulfite converted using an EZ DNA Methylation Gold kit (Zymo Research). After PCR amplification of approximately 200 bp regions encompassing the target loci using specifically designed primers to ensure unbiased amplification, quantitative measurement of DNA methylation at each CpG was performed using a pyrosequencing primer located within 30 bp of the CpG interrogated. Reactions were measured on a PyroMark Q96 MD Pyrosequencer following the manufacturer's protocol, and analyzed using the Pyro Q-CpG software (Biotage, Uppsala, Sweden), which allows quality assessment of each measurement. CpG loci that were called 'passed' in the default software settings are shown in Figure 1b (*n *= 175 for *IGSF2*; *n *= 156 for *PLSCR2*). To assess the agreement between methods, we used Pearson's correlation (as throughout the manuscript), because rank-based correlations do not account for the clustering of most samples within a small range of methylation (for example, 95 to 100% methylation for *PLSCR2 *in Figure 1b). An alternative metric, classifying sites into high or low methylation based on a cutoff and measuring agreement in a 2 × 2 contingency table, led to results similar to the Pearson correlation across a wide range of cutoffs (data not shown). Primer sequences used for DNA amplification and pyrosequencing are available upon request.

### Calculation of false discovery rates

All FDRs were estimated by randomization, which preserves all aspects of the data that might affect statistical analyses. For example, the FDR for population-specific methylation was estimated by randomly assigning CEU/YRI labels, and recalculating the Wilcoxon *P*-value on the randomized data (resulting in an essentially uniform distribution of *P*-values, like that shown in Figure 1c). FDRs for mSNPs were estimated by pairing genotypes with randomly chosen methylation profiles, and calculating mSNPs as for the real data. Because of the family trio structure of the HapMap samples, not all samples are independent; to account for this in our randomization procedure, we also performed randomizations based on swapping methylation data for entire trios, in effect treating each trio as an independent unit composed of three methylation profiles and three genome sequences. This procedure yielded indistinguishable FDRs compared to randomizing all samples individually. All FDRs are based on at least 1, 000 randomizations.

### Heritability analysis

Narrow-sense heritabilities (*h*^2^) were estimated as the correlation between average parental values and their offspring. Because the offspring and parental variances are equal, this is equivalent to performing regression. Although heritabilities are by definition non-negative, our estimates are often negative due to the limited power inherent in our data. We note that our method of estimating *h*^2 ^assumes that there is no shared environmental variance between parents and offspring that impacts DNA methylation; if this assumption is violated, we will overestimate *h*^2 ^(with an upper bound of *H*^2^, the broad-sense heritability). It also assumes that somatic DNA methylation is not passed directly from parent to offspring through the germline, since this would violate the assumptions of the heritability estimation. To estimate the number of CpG sites with heritable methylation, we generated 1, 000 randomized versions of the *h*^2 ^distribution (see above), and calculated the number of sites with greater methylation in the real data, compared to each randomized distribution. Visually, this corresponds to the area in between the two distributions, on the right side (positive values) where the real distribution is shifted to the right. The average difference across the 1, 000 randomizations was 762 sites for CEU, and 930 for YRI. Note that this procedure allows us to estimate the number of heritable sites, but not specify which specific sites are the heritable ones; thus, it is not possible to calculate an FDR for these estimates.

### mSNP analysis

mSNPs were identified by calculating correlations between SNP genotypes (arbitrarily coded as 0, 1, and 2) and methylation levels. Only SNPs within 100 kb of each CpG site were tested, to reduce the multiple testing burden. Although the 1000 Genomes SNP catalog is more complete, we used HapMap genotypes [16] for the mSNP analysis, since not all cell lines for which we collected methylation data have been sequenced as part of the 1000 Genomes Project [22]. We required a minimum of 5 minor alleles among the 90 individuals of each population to include a SNP in this analysis (for details of how we accounted for the family trio structure, see 'Calculation of false discovery rates' above). This resulted in 2, 668, 982 YRI SNPs and 2, 405, 735 CEU SNPs (1, 969, 973 shared by both). For the analysis of genetic variants contributing to population-level differences, only the SNPs shared by both populations were used, and population was represented in the multiple regression as 0/1 for CEU/YRI.

Correlations were recorded as the absolute value of the correlation coefficient, since the sign is arbitrary, depending on how genotypes are coded as 0/1/2. However, for comparisons between CEU and YRI correlations, the fact that all correlations are positive means that the difference between associations can be underestimated. If the same SNP (or two SNPs in high LD) was used to calculate the correlation with a particular CpG site's methylation in both populations, the signs could be used; however, in most cases a site's strongest correlation was with different SNPs in CEU and YRI, precluding the use of signs.

### Bisulfite sequencing of *RNF186 *promoter region

Genomic DNA (500 ng) was bisulfite converted using the EZ-96 DNA Methylation Gold Kit (Zymo Research) as per the manufacturer's protocol with minor modifications. A 532 bp region upstream of the *RNF186 *gene containing the SNP rs3806308 and the CpG site cg09195271 from the IlluminaHuman Methylation array was amplified by nested PCR reactions using Hotstar Taq (Qiagen, Hilden, Germany). The first round of PCR amplification was done using 55°C annealing temperature for 30 cycles and the primer pair F3 (GGATATAGAGGGTGGTTTGTAGTGTTAGT) and R2 (ACRCACAAATATTTAACACCTACTACT). A 3 μl aliquot of the material obtained in the first round was further amplified in the second round in a total volume of 50 μl, using 51°C annealing temperature for 35 cycles and the primer pair F2 (TGAATGAAATATTTGTTTGAGGGAGTGT) and R3 (CCTTAAAACCACAACTATTATATTCACAA). All primers were designed to be specific for bisulfite converted DNA. The amplified PCR product was separated from primers by electrophoresis in a 1.5% Tris-acetate-EDTA (TAE) agarose gel, excised and purified using the QIAquick gel extraction kit (Qiagen). Purified DNA was then ligated into plasmid pGem-T Easy using the pGem-T Easy vectory system (Promega, Madison, WI, USA) and transformed into competent JM109 *Escherichia coli *(Promega) by the CaCl_2 _method. Colonies carrying a plasmid containing an insert were then selected based on blue-white screening. Plasmid DNA was extracted using Qiaprep Spin Miniprep kit (Qiagen). Plasmid clones containing the appropriate sized insert, as determined by a restriction digestion analysis, were sequenced using T7 and/or SP6 primers by Genewiz Inc. South Plainfield, NJ, USA. Sequences were analyzed using Sequencher sequence analysis package 4.6 (Gene Codes Corporation, Ann Arbor, MI, USA).

## Abbreviations

bp: base pair; CEU: HapMap population of Northern European ancestry; CpG: cytosine-phosphate-guanine; FDR: false discovery rate; GxE: gene-by-environment; GxG: gene-by-gene; LCL: lymphoblastoid cell line; LD: linkage disequilibrium; mSNP: methylation-associated SNP; SNP: single-nucleotide polymorphism; YRI: HapMap population of Yoruban ancestry.

## Competing interests

The authors declare that they have no competing interests.

## Authors' contributions

MSK designed the project, oversaw data generation and wrote the paper. HBF designed the project, analyzed the data and wrote the paper. LL and SN generated and normalized the data. All authors have approved the final manuscript for publication.

## Supplementary Material

Additional file 1 **Supplemental text, Tables S1 and S2, and Figures S1 to S19 **[27-30]. Click here for file
