# Supplementary figures and images for: Population-specificity of human DNA methylation
Source: Genome Biol. 2012 Feb 9;13(2):R8. doi: 10.1186/gb-2012-13-2-r8 (PMC3334571; doi:10.1186/gb-2012-13-2-r8)

Figure S1

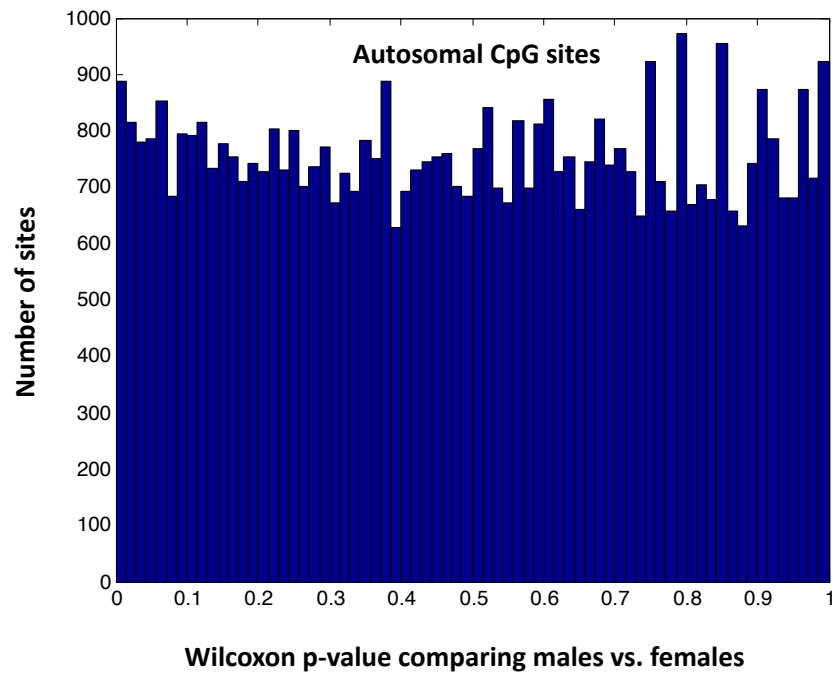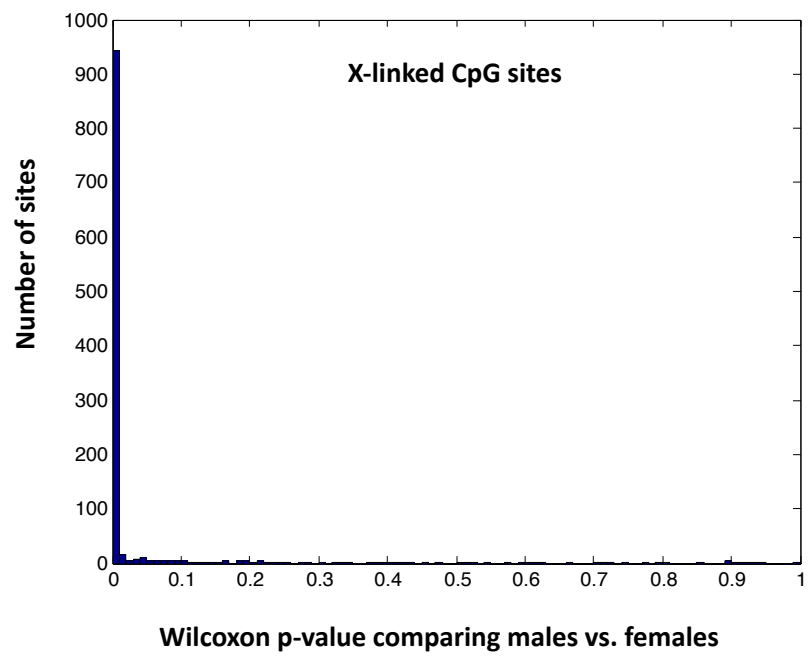

Figure S2

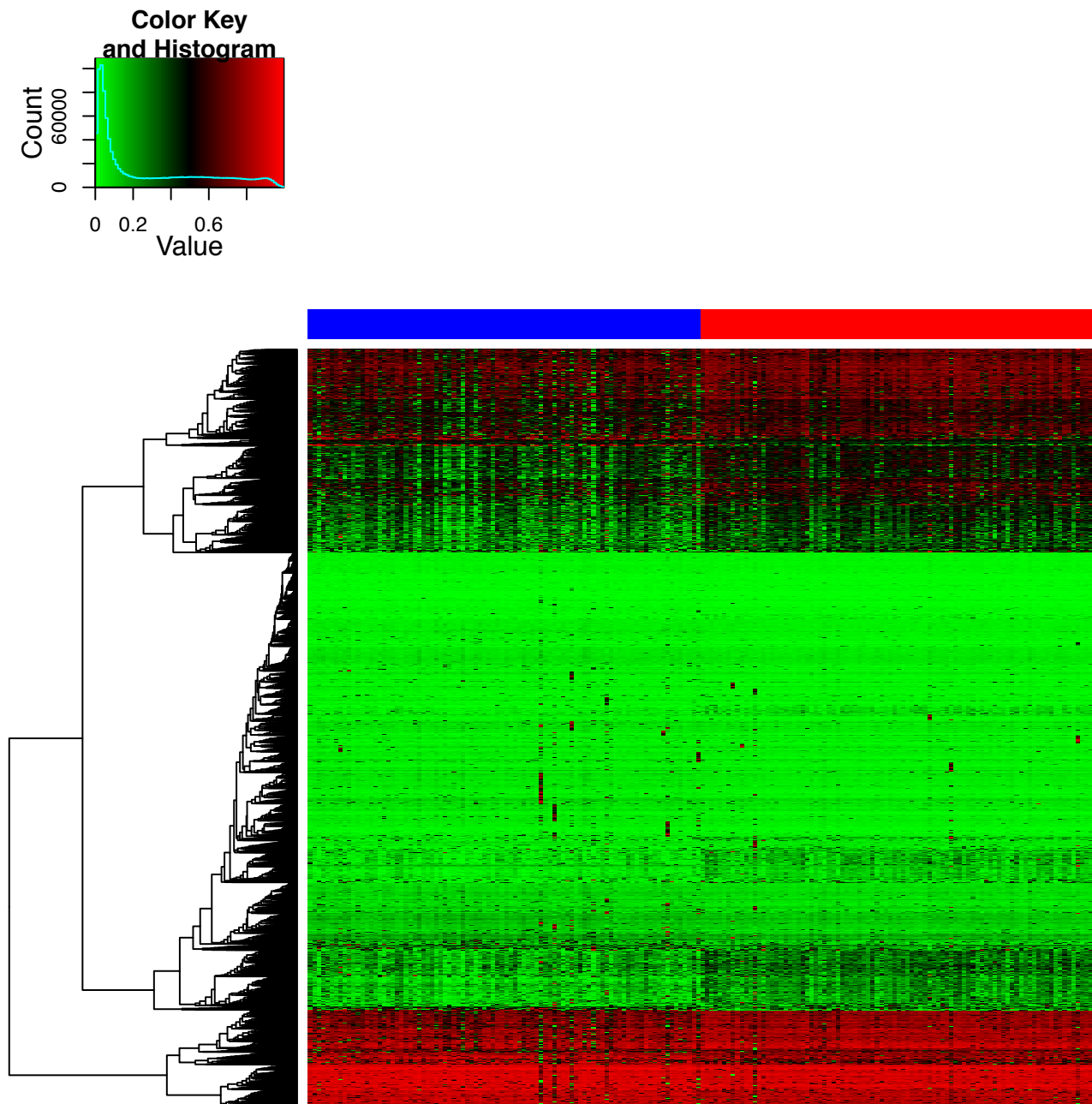

Figure S3

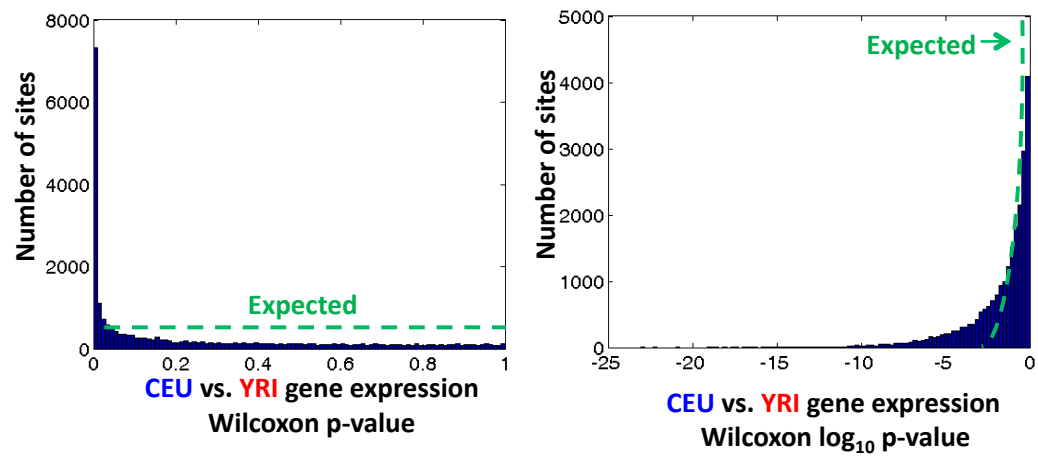

Figure S4

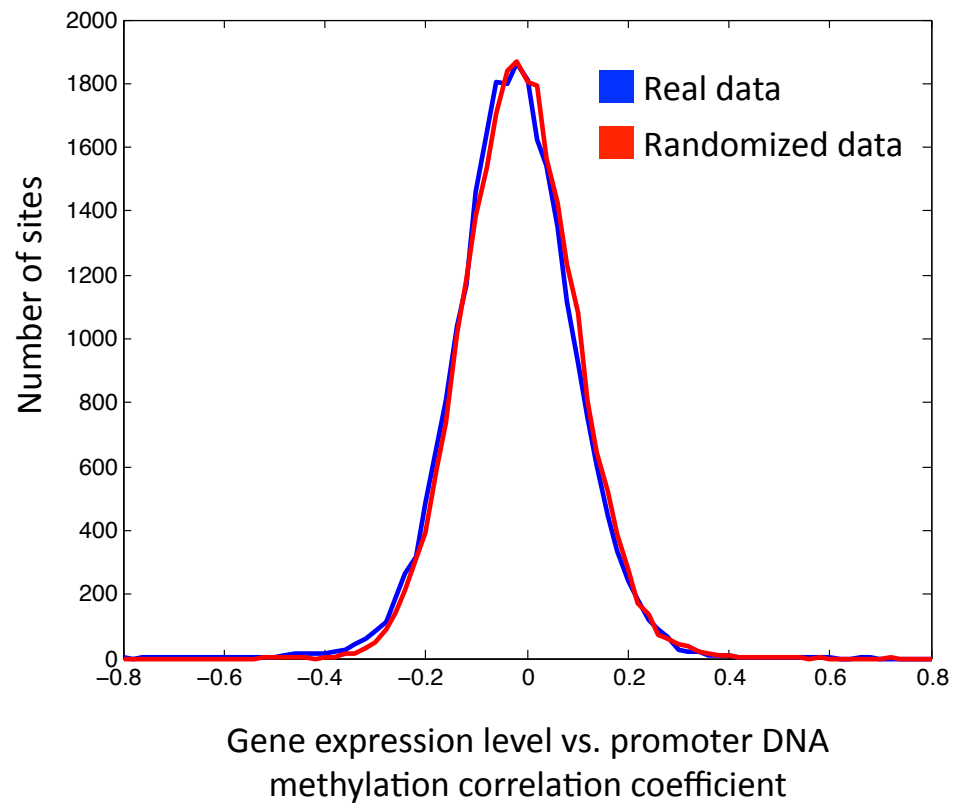

Figure S5

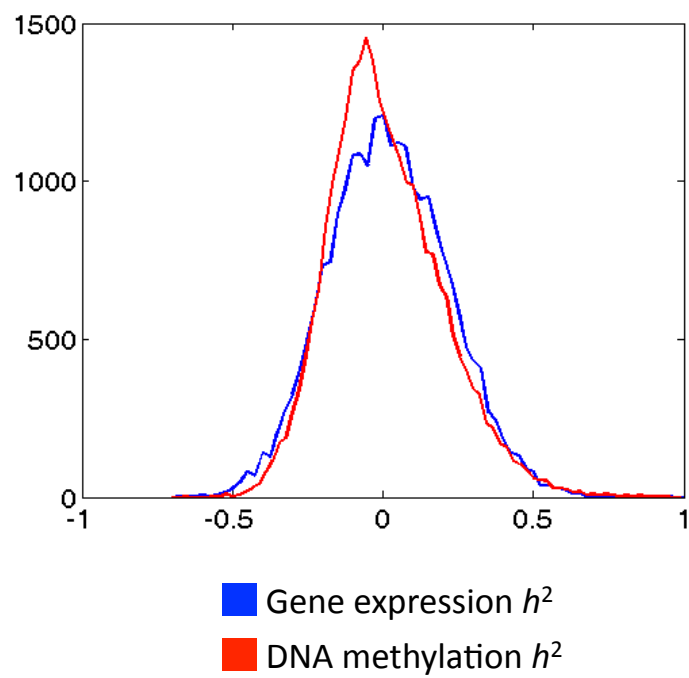

Figure S6

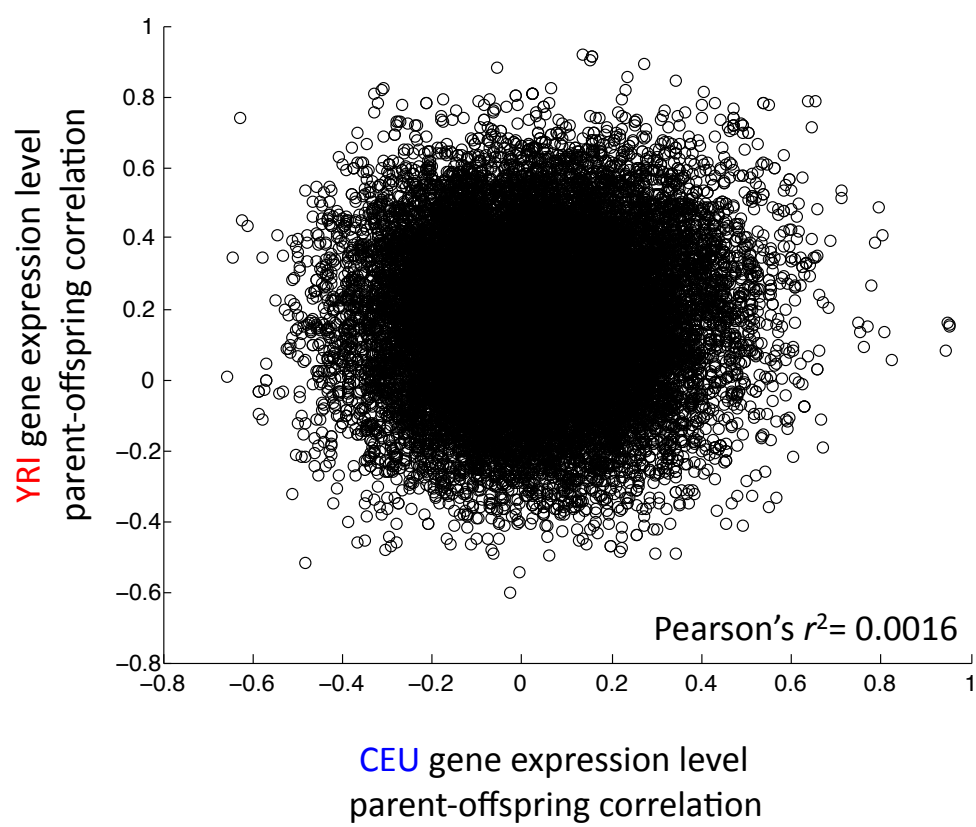

Figure S7

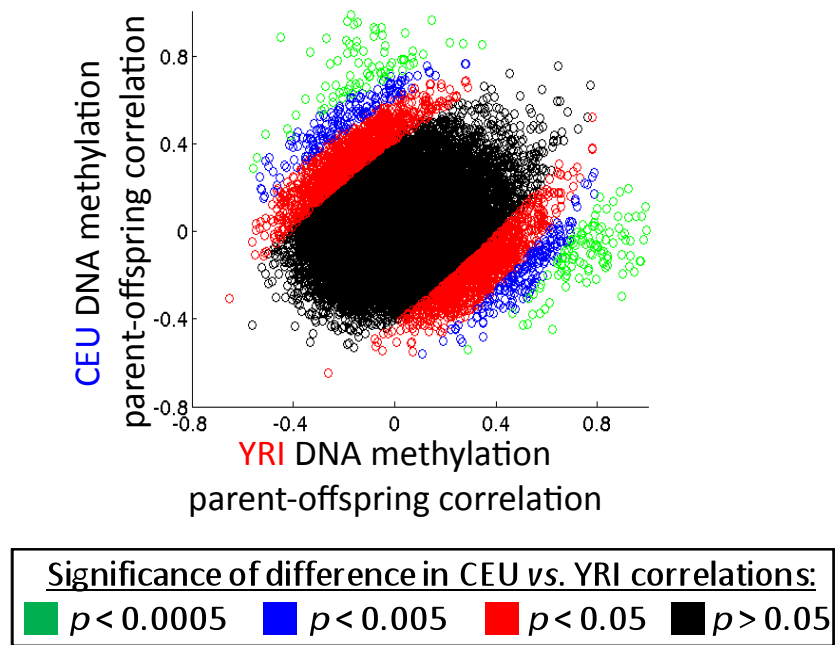

Figure S8

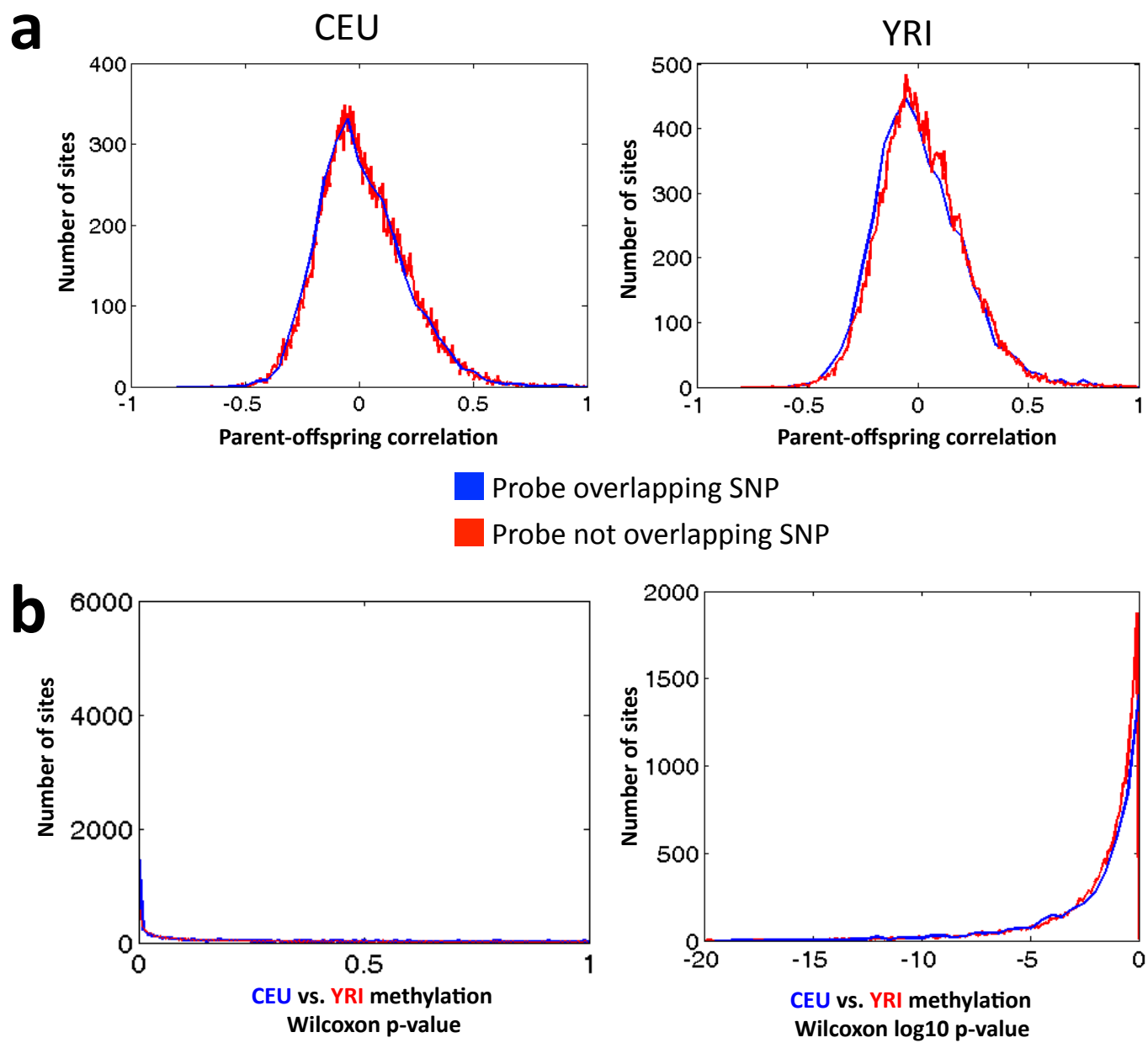

Figure S9

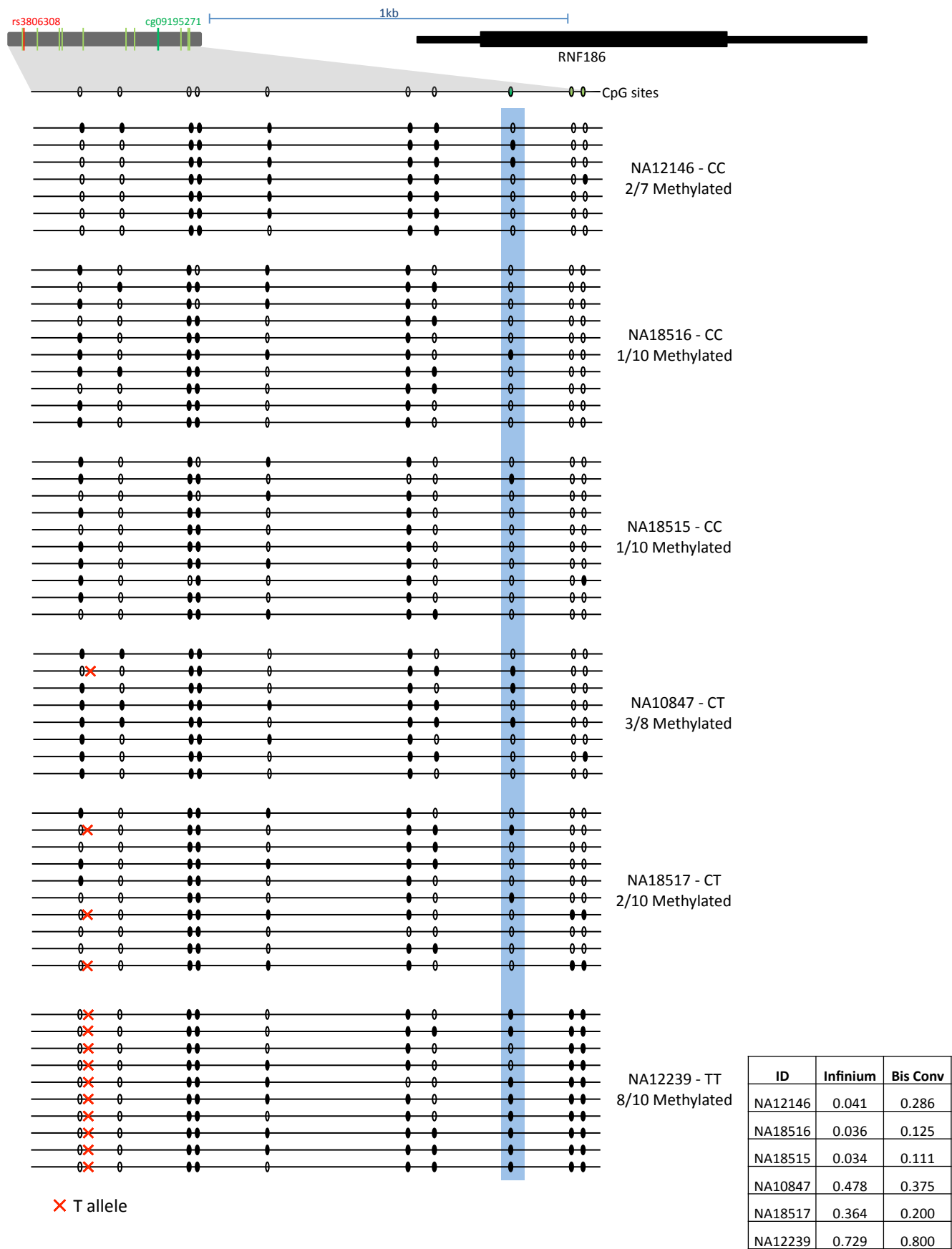

Figure S10

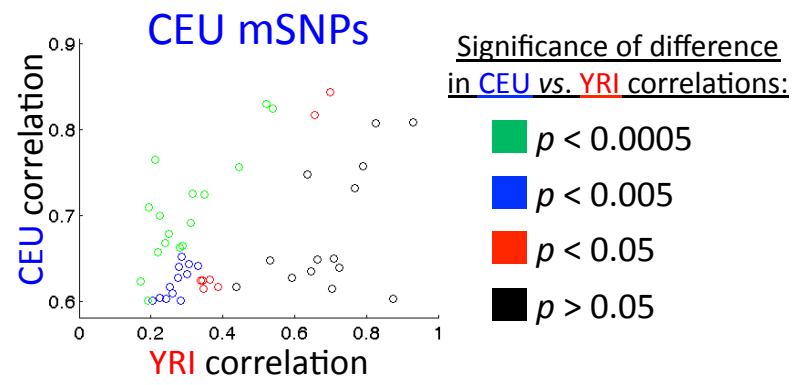

Supplement: Additional file 1 — Supplemental text, Tables S1 and S2, and Figures S1 to S19 [27-30]. [file gb-2012-13-2-r8-S1.ZIP › supp figs.pdf]
