# Supplementary material for: Population-specificity of human DNA methylation
Source: Genome Biol. 2012 Feb 9;13(2):R8. doi: 10.1186/gb-2012-13-2-r8 (PMC3334571; doi:10.1186/gb-2012-13-2-r8)
Supplement: Additional file 1 — Supplemental text, Tables S1 and S2, and Figures S1 to S19 [27-30]. [file gb-2012-13-2-r8-S1.ZIP › supplemental text.doc]

**Age as a potential confounding factor**

DNA methylation patterns in some tissues are known to be age-dependent [14, 27], but this effect has not been reported in cell lines (to our knowledge). To test whether age may be a confounding factor in our analysis, we collected all available data for ages of the HapMap participants at the time of blood draws. This consists of ages for 63 CEU individuals (median = 67 y; range = 37 – 97). No age data for YRI were available. These ages showed no more correlation with the methylation of individual CpG sites than expected by chance, nor did they correlate with the principal components of the entire data set more than expected (data not shown). Thus we conclude that age is not an important confounding factor in our analysis, though we cannot rule out the possibility of weak correlations with methylation at a small number of CpG sites.

**Comparison of DNA methylation with gene expression variation**

Consistent with previous results [13], we observed very little correlation between the methylation state of a gene’s promoter and its expression level across individuals, as shown in Fig S4 (This lack of correlation should not be confused with the well-established relationship of DNA methylation levels with gene expression across genes, within an individual.). Also similar to Bell et al. [13], we found only a small overlap between mQTLs and eQTLs in our data: for instance six of the 55 high-confidence YRI mQTLs were previously identified as eQTLs in these same cell lines [28-29]. Therefore it appears that the methylation states of the CpG sites we studied do not play a major role in determining mRNA levels in LCLs. It remains possible that variation in DNA methylation could predominantly affect tissue-specific gene expression, in which case measuring expression in any single cell type would show little overall relationship; or that DNA methylation may affect the dynamics or inducibility of expression, not captured by steady-state mRNA level measurements; or that methylation of CpGs other than those examined here are most relevant for determining expression levels.

**Differences between CEU and YRI methylation heritability**

As stated in the main text, the difference between the heritabilities of YRI and CEU CpG methylation could be due either to true differences, or low power. To differentiate between these, we tested whether the heritabilities within each population were significantly different from one another—which could not result from limited power. For this analysis we required a null hypothesis of equal heritabilities that, when rejected, would yield evidence of a significant difference (instead of simply a lack of correlation, shown in Fig 2c, which could be caused by inaccurate heritability estimates). To achieve this, we estimated the probability that each site’s midparent-offspring correlations were consistent with a single underlying magnitude. We first performed Fisher’s *r*-to-*z* transformation (*z* = ln([1 + *r*]/[1 – *r*])/2), and then computed the statistic (*z*_1_ – *z*_2_)/√(2/(*n* – 3)), which is normally distributed with zero mean and unit standard deviation (*n* = sample size of each correlation, which is 30 in this case). In this test, low power due to noisy data or limited sample size would only make it more difficult to achieve significance beyond what is expected by chance. However we were unable to reject the null hypothesis of low power, since the number of significant differences was close to that expected by chance (data not shown).

**Dominance, maternal, and paternal effects**

In conjunction with the heritability analysis, we tested for several other forms of inheritance of DNA methylation. For a trait determined by additive genetic variation, the expected offspring value equals the average of the two parents. Other forms of inheritance could have a bias in which parent’s methylation determines the offspring’s—such as the mother’s only, the father’s only, the parent with greater methylation only, or the parent with the lesser methylation only. For each CpG site, we tested all four of these alternative models, while choosing either the mother or father randomly as a control. We found no evidence for any of the four potential modes of inheritance—i.e., their predictive value was no greater than the random parent control, and was less than that of the parental average (data not shown).

**Effects of excluding probes containing SNPs**

As discussed in the main text, probes containing SNPs were significantly enriched for those with apparent mSNPs, so for this reason were excluded from that analysis. In contrast they did not show any difference in terms of population differences in DNA methylation levels, or in heritabilities (Fig S8). Specifically, after excluding the 4,868 autosomal probes with a SNP in either population, we found 4,512 / 21,618 = 20.9% of CpG sites to be differentially methylated between populations at Wilcoxon p = 0.001, and 6,840 / 21,618 = 31.6% at p = 0.01. The number of CpG sites with heritable methylation (as defined in the Methods) decreased from 762 / 26,486 (2.9%) to 607 / 21,618 (2.8%) in CEU, and from 930 / 26,486 (3.5%) to 730 / 21,618 (3.4%) in YRI. The correlation between *h*^2^ estimates across all sites not overlapping SNPs was *r*^2^ = 0.003. Excluding probes with potential cross-hybridization to other genomic regions [30] had similarly minor effects.

**Testing trivial explanations for population-specific mSNPs**

We considered three possibilities to explain our observation of population-specific mSNPs: low power, differing LD, and differing allele frequencies.

To test low power as an explanation, we calculated for each mSNP whether the association strength observed in each population was consistent with a single underlying genetic effect; while low power could result in our detecting mSNPs in only one population, it could not lead to significantly different effect sizes. To compare correlation coefficients, we performed Fisher’s *r*-to-*z* transform, as explained above for the heritability difference analysis (with *n* = 90). Using this test, we found that most mSNPs (79% of YRI and 73% of CEU mSNPs; Fig 3d and Fig S10) were not consistent with a single genetic effect size. This result by itself is not enough to rule out the possibility of low power, however, because our mSNPs represent a highly biased subset of associations (namely, the strongest in one cohort/population). The Beavis effect, or “winner’s curse”, can lead to significant differences in effect sizes in a second cohort when replicating the strongest hits from the original cohort, so we sought to test if this could account for the differences we observed. If we were testing only one SNP per CpG site, then under the null hypothesis of equal effect sizes, our *p*-values (assessing the probability of observing correlations as different as they are, under the null) would be uniformly distributed over [0,1]. Thus when testing 26,486 CpG sites, we would expect ~132 associations differing at the *p* < 0.005 level (this is 26,486 x 0.005). However, our analysis does not fit this simple model. We test many (average ~200) SNPs per CpG site, and then use the correlation with maximum absolute value in each population to represent that site’s association strength. Taking the maximum absolute value over hundreds of data points compresses the distribution of correlation coefficients substantially, and therefore lowers the expected difference between the two populations. To assess the extent of this compression, we conducted 100 independent simulations of methylation at 26,486 CpG sites in 90 individuals, while varying the number of SNPs tested per site, their LD structure, and the true association strength. Across all simulated associations, we did not observe any correlations differing between populations even at the *p* < 0.005 level (as a result of the compression mentioned above). Since most of the mQTLs in the real data differ between populations at *p* < 0.005, we conclude that the Beavis effect is unlikely to explain our results.

Because most mSNPs are not expected to be the causal variants, but instead are proxies in LD with these variants, differences in LD between populations are expected to result in different mSNPs, even when the causal variant is unchanged. In contrast, comparing the methylation sites associated with mSNPs (Fig 3b) is much less susceptible to differences in LD, but may still be affected if all of one population’s mSNPs (both causal and tag SNPs) for a particular CpG site were not genotyped in the HapMap. To test this possibility, we re-tested all population-specific associations with a nearly complete catalog of common genetic variants (from the 1000 Genomes Project) in these cell lines. We found no additional CpG sites’ methylation associating with SNPs in this essentially complete set of variants, suggesting that differing LD also cannot account for the population-specificity of mSNP associations that we observed.

The final trivial explanation for population-specificity is that an mSNP could be at too low a frequency in one population to be detected. This is indeed the case for about one-third (36%) of mSNPs, which are common in one population but rare (<5% minor allele frequency) in the other. However the remaining 64% are common in both populations, leaving the majority of the population-specificity unexplained.

**Comparison with Bell *et al*. (2011)**

A recent study [13] performed mQTL analysis on 77 YRI LCLs, including 60 of the same ones included in our study. The use of the same methylation bead-array platform makes comparison between the two straightforward. Among our 55 high-confidence YRI mSNPs, 19 (34.6%) were also identified as targets of mSNPs by Bell et al. Given the total number of autosomal methylation probes on our arrays, this overlap is ~50.8-fold higher than expected by chance. Reasons for the ~65% not overlapping could include use of different methodologies for identifying mSNPs (e.g. distance searched from each CpG site), use of different YRI individuals, and low power.

**Supplemental Figure and Table legends**

**Table S1. Infinium probes overlapping SNPs from 1000 Genomes Project samples.**

**Table S2. High-confidence mSNPs from YRI.** See Table 1 for explanation.

**Figure S1. Gender differences in DNA methylation.** The Wilcoxon test was performed to compare DNA methylation levels at all sites between males vs. females. In the autosomal sites (top), the p-values are uniformly distributed, indicating no sex-specific methylation. In the X-linked sites (bottom), nearly all sites show extremely strong sex-specificity, as expected because of the highly methylated inactive X-chromosome in females.

**Figure S2. Heatmap of population-specific CpG methylation.** Heatmap is the same as Fig 1a, except only for the 5,654 sites differing between populations at Wilcoxon *p* < 0.001. The histogram indicates the distribution of average methylation values for these 5,654 sites.

**Figure S3. Population-specificity of gene expression levels.** Gene expression levels were measured using Affymetrix Exon arrays in 176 of the 180 cell lines studied here [20]. We applied the Wilcoxon test, comparing the expression levels of RefSeq genes in CEU vs. YRI. 31.1% of genes have *p* < 0.01, and 19.6% have *p* < 0.001 (compared to 32.0% and 21.4% for DNA methylation at the same cutoffs).

**Figure S4. Comparison of DNA methylation and gene expression levels across individuals.** Little difference in the distribution of correlations is observed, compared to randomized controls. See section “Comparison of DNA methylation with gene expression variation”.

**Figure S5. Heritability of gene expression levels.** Using the same gene expression data as in Figs S3-S4 [20], we estimated the heritability of each expression level by its midparent-child correlation (as shown for methylation levels in Fig 2).

**Figure S6. Comparison of YRI and CEU gene expression heritabilities.** Similar to Fig 2c, except for gene expression levels [20] in place of DNA methylation.

**Figure S7. Significance of heritability differences.** We calculated the probability that each site’s pair of heritability estimates could have arisen from a single underlying effect size, using Fisher’s *r*-to-*z* transformation (see Methods), and coloring each data point by its *p*-value.

**Figure S8. Methylation heritabilities and population-differences for probes overlapping SNPs. a.** Distributions of *h*^2^ estimates for probes overlapping SNPs (Table S1), and those not overlapping SNPs (The histograms for probes not overlapping SNPs are less smooth because the number of bins was increased, to make the average number of probes per bin equal to the histogram for SNP-containing probes.). The similarity of the histograms indicates that SNPs in probes do not appreciably affect our heritability estimates (see section “Effects of excluding probes containing SNPs”). **b**. Distributions of Wilcoxon *p*-values assessing population differences, for probes overlapping SNPs (Table S1), and those not overlapping SNPs (as in Fig 1c). The similarity of the histograms indicates that SNPs in probes do not appreciably affect our analysis of population-level differences in DNA methylation.

**Figure S9. Bisulfite sequencing of the *RNF186* promoter region.** (Top) Schematic diagram of the *RNF186* gene, relative to sequenced region. Grey box indicates region sequenced which is 600bp upstream of the *RNF186* gene, including the SNP rs3806308. Clones containing the T allele of this SNP are indicated by a red X (note that the T allele disrupts a CpG site shown adjacent to the SNP, so these cannot be methylated). The CpG site assayed by the Illumina HumanMethylation27 array (cg09195271) is highlighted in light blue. Each line is one clone, which represents a single DNA molecule. Filled ovals representing methylated CpG sites and open ovals representing unmethylated CpG sites. The methylation at CpG site cg09195271 is associated with the rs3806308 genotype, even though six other CpG sites closer to the SNP show no association (two additional CpG sites to the right of cg09195271 have methylation that is weakly associated with the genotype). Ratio of methylation refers to number of clones that were methylated at cg09195271 divided by the number of clones sequenced for each sample. The percentage of this ratio is indicated in table under “Bis Conv”. Infinium indicates the methylation level of cg09195271, in average beta (percentage methylation), as determined by the Illumina HumanMethylation27 array. Pearson correlation between bisulfite sequencing determined methylation status with Infinium determined methylation is 0.863.

**Figure S10. Population-specificity of CEU mSNPs.** As for Fig 3d, but with CEU mSNPs.
